# Development of Virtual Mental Health Stepped Care Service for a Heart Failure Remote Management Program: Qualitative Descriptive Study

**DOI:** 10.2196/82139

**Published:** 2026-04-14

**Authors:** Amika Shah, Anam Shahil-Feroz, Kathleen A Sheehan, Shannon Wright, Robert P Nolan, Gillian Strudwick, Sanjeev Sockalingam, Emily Seto

**Affiliations:** 1 Institute of Health Policy, Management and Evaluation, Dalla Lana School of Public Health, University of Toronto, 155 College St 4th Floor, Toronto, ON, M5T 3M6, Canada, 1 (416) 978 4326; 2 Centre for Digital Therapeutics, University Health Network, Toronto, ON, Canada; 3 Arthur Labatt Family School of Nursing, Faculty of Health Sciences, The University of Western Ontario, London, ON, Canada; 4 University Health Network, Toronto, ON, Canada; 5 Department of Psychiatry, University of Toronto, Toronto, ON, Canada; 6 Lawrence Bloomberg Faculty of Nursing, University of Toronto, Toronto, ON, Canada; 7 Cardiac eHealth, Toronto General Hospital, University Health Network, Toronto, ON, Canada; 8 Institute of Medical Science, University of Toronto, Toronto, ON, Canada; 9 Centre for Addiction and Mental Health, Toronto, ON, Canada

**Keywords:** telemedicine, e-counseling, patient care management, heart failure, digital health, telemonitoring, remote monitoring, depression

## Abstract

**Background:**

Depression is highly prevalent yet undertreated among people living with heart failure, indicating barriers to mental health services. Although various digital mental health interventions have been developed to detect, treat, and manage depression in this population, these interventions have seen limited integration into clinical care and a lack of implementation research. Stepped care is a service innovation that may promote the implementation of these technologies into clinical settings, but few studies have examined how these services are designed in clinical settings.

**Objective:**

This study aimed to identify strategies to address health system barriers to accessing mental health care from the perspective of people living with heart failure, clinicians, and researchers, and to incorporate these strategies into the design of a virtual mental health stepped care service within a heart failure remote management program.

**Methods:**

A qualitative description study was conducted using purposive recruitment of people living with heart failure, clinicians, and researchers from a heart failure remote patient management program. As part of a service design approach, semistructured interviews explored potential strategies to address barriers to accessing mental health services. Two researchers coded the data descriptively and constructed themes to guide the development of a virtual stepped care service.

**Results:**

A total of 22 participants were interviewed, comprising 13 people living with heart failure and 9 clinicians and researchers. Six themes were identified, comprising 4 requirements and 2 foundational principles. The requirements were to (1) adopt a collective approach to identify distress across methods, people, and time points; (2) maintain a referral-based approach; (3) rely on existing mental health human resources; and (4) offer patient choice among various mental health care options. These requirements were supported by two principles: (1) building on organizational strengths and (2) reducing treatment burden. Based on these findings, a virtual stepped care service was developed, incorporating a depression screening module, referral-based workflows, and, where clinically appropriate, patient choice in treatment selection.

**Conclusions:**

The stakeholder-informed design of this virtual stepped care service contributes to the limited literature on stepped care service design and demonstrates how such models can be tailored to their intended contexts. Although each component was designed to address health system barriers to mental health care for people living with heart failure, resource limitations may constrain the balance between feasibility and quality of care. Future research should evaluate the acceptability of this model among people living with heart failure and clinicians.

## Introduction

### Background

Despite the established impact of depression on heart failure outcomes, this prevalent comorbidity remains unaddressed or underaddressed in usual care [1,2]. Clinical practice guidelines, in addition to a growing body of research, have called for a more integrated approach to addressing depression, such as incorporating depression screening and diagnosis into heart failure care [1,3-5]. However, in prior qualitative research, our research team identified that this remains hindered by barriers at the health system and patient levels [6] and may contribute to the poor uptake of mental health services documented within this population [7-9].

In both the heart failure and mental health service literature, virtual technologies are seen as promising tools to enhance access to mental health services [1]. Virtual mental health, as defined by Jeunemaître, encompasses “technological applications used for mental health assessment, support, prevention, and treatment” [10]. Despite this potential and the effectiveness of virtual mental health interventions in controlled research settings for people living with complex chronic conditions, their uptake and integration into real-world clinical care have remained limited [11-13]. Scholars argue that closing this research-to-practice gap is a critical priority to improve population-level outcomes for those at heightened risk, such as people living with heart failure [11].

To foster the adoption of virtual mental health technologies into clinical care, scholars have pointed to a narrow and often simplistic view of what constitutes a virtual mental health intervention as a key barrier to their implementation [11]. In proposing three critical misconceptions facing the field of virtual mental health, Mohr et al [11] argue that the prevailing view of virtual mental health technologies as stand-alone products (ie, a focus exclusively on the technology itself) fails to account for the broader context in which these technologies are deployed. Instead, they suggest a move away from viewing these technologies as mere products toward viewing them as technology-enabled services, encompassing the people, workflow, and organizational contexts that shape their implementation. This recommendation has been echoed more broadly by virtual health scholars [11,14-16]. Taken together, the existing literature suggests that successful implementation of virtual mental health technologies requires not only product innovation, but also service innovation*,* with active engagement from stakeholders essential in designing both aspects [14-16]. Yet, while there is ample literature on virtual mental health product innovation, the literature on service innovation surrounding these technologies has been comparatively scarce [17].

Stepped care is an often-discussed service innovation to promote the implementation of virtual mental health technologies for those living with chronic conditions [12]. Traditionally, stepped care is defined by two core principles: (1) individuals should be given the least restrictive mental health service first, and (2) patients should “step up” to more intensive services as needed [18]. Since the emergence of stepped care, various architectures have been explored, often varying based on how treatment is allocated to the patient. For example, “stratified” or “matched” architectures determine treatment intensity by assessing the severity of the patient’s symptoms [19]. In contrast, the Stepped Care 2.0 architecture has challenged this stratified approach, opting to move away from organizing interventions in a hierarchy [20,21]. Instead, Stepped Care 2.0 emphasizes a more patient-centered approach to care allocation, with less reliance on diagnostic assessment and greater inclusion of patient preference [20]. Although there has been growing interest in various types of stepped care models to implement virtual mental health technologies, how these models should be designed with the perspectives of diverse stakeholders in the implementation context remains unclear. This is particularly important as research has suggested that stepped care should be tailored to the local contexts and the available resources in these settings, though few studies have explicitly described the process of doing so for clinical settings outside postsecondary institutions [22].

### Objective

The objectives of this study were twofold: (1) to explore strategies to address health system barriers to accessing mental health services for patients enrolled in a heart failure remote management program, and (2) to develop a virtual mental health service that implements these strategies to improve access to mental health care for people living with heart failure. The research question guiding this study was “How should a virtual mental health stepped care service be designed to improve access to mental health care for people living with heart failure based on the perspectives of individuals living with heart failure, clinicians, and researchers?”

## Methods

### Overview

A service design approach was used to develop the virtual stepped care service for people living with heart failure [15]. Service design is a “human-centered, collaborative, interdisciplinary, iterative approach which uses research, prototyping, and a set of easily understood activities and visualization tools to create and orchestrate experiences that meet the needs of the business, the user, and other stakeholders” [23]. Through understanding touchpoints (when an individual engages with the organization) and the journey (a series of touchpoints), service design endeavors to create services centered upon the experience of people who engage with them [23].

### Objective 1 (Methods)

To address the first study objective of exploring strategies to overcome system-level barriers to accessing mental health services for people living with heart failure, qualitative interviews were conducted with stakeholders at the University Health Network, Toronto General Hospital, to identify key barriers (pain points) people living with heart failure face when seeking mental health services and to identify potential strategies to address these pain points. As the purpose of this study was to improve heart failure care through collecting rich descriptions of participant perspectives, this research was situated within a naturalistic approach, which assumes that reality is subjective and experienced differently by each individual (relativist ontology), and that knowledge is shaped by those experiences (subjectivist epistemology) [24,25]. A qualitative description methodology, as outlined by Sandelowski [25], was selected to understand stakeholder perspectives on the design of a virtual mental health stepped care service so that they could be incorporated into the design of a new service [26].

Qualitative description was chosen for its focus on staying close to the data when exploring practice- and policy-relevant questions. This approach aims to understand and ascribe meaning to the phenomenon (the design of a virtual mental health stepped care service for a heart failure remote management program) as closely as possible to the participants’ perspectives to support descriptive and interpretive validity, respectively [25,27]. In the case of this research, a description of the requirements of a new service was desired to ensure that the needs of the stakeholders who would be using, involved with, and affected by the newly developed service delivery model were addressed. Moreover, the focus on understanding and interpreting phenomena as closely as possible to the study participants was especially valuable given the early stages of the service design process, wherein the goal is to prioritize the views and needs of participants in the design of virtual health services.

#### Theoretical Framework

To explore how a virtual mental health stepped care service could be designed to improve access to mental health care, we drew upon the patient-centered access to care framework proposed by Levesque et al. [28]. This framework was selected for its conceptualization of access as a journey, rather than a single event, composed of 5 stages: perceiving a need or desire for health care, seeking health care, reaching services, using those services, and experiencing consequences of health care services (positive or negative) [28]. Within each stage of this journey, factors at both the supply side (eg, health care system) and demand side (eg, patients and their support networks) may facilitate or prevent health care access. On the supply side, these include the approachability, acceptability, availability, accommodation, affordability, and appropriateness of health care services. These are complemented by factors on the demand side, namely, the ability of patients and their support networks to perceive a need for care, seek health care services, reach services, as well as pay for and engage with health care services.

In a previous study using this framework, our research team identified barriers faced by people living with heart failure when accessing mental health care in Ontario, Canada, using Levesque’s framework [6]. Table 1 summarizes the domains of access at each stage of seeking health care, as well as the barriers found to prevent people living with heart failure from accessing mental health care identified in this previous investigation. The current study builds upon this previous research by describing the barriers experienced by people living with heart failure during their journey of accessing mental health services and applies Levesque’s framework to inform the design of the virtual mental health stepped care service. Specifically, the framework was used to determine how each stage of access and associated health system barriers may be intentionally addressed by potential features of a virtual stepped care service [28].

**Table 1.** Health system barriers impacting access to mental health services for people living with heart failure.

Stage of access [28]	Health system factors impacting access [28]	Health system barriers faced by people living with heart failure [6]
Perceiving health needs and desire for care	Approachability	Difficulties detecting mental health concerns Unpreparedness for referral conversations
Seeking health care	Acceptability	—^a^
Reaching health care	Availability and accommodation	Limited types of mental health services available Inconsistent pathways to mental health services Poorly timed mental health services
Using health care	Affordability	Limited human resources due to underinsurance of mental health care
Health care consequences	Health care consequences	An underresourced system does not allow for choice or finding a fit Insufficiency of generic mental health services

a Not available.

#### Setting

Individuals living with heart failure were recruited by phone from the Medly (University Health Network) program, which has been operational as standard of care at Toronto General Hospital, a large urban hospital in Ontario, Canada, since 2016. The Medly program is a smartphone-based heart failure remote patient management program that supports clinical care and patient self-management [29]. The Medly smartphone app is available on iOS and Android and prompts patients to enter their blood pressure, weight, heart rate, and symptoms regularly [29]. Depending on their entries, patients receive automatic self-care messages based on an alert system. Should patients score within a concerning range, their clinical care team is notified via email alerts and a clinician dashboard, who provide follow-up care based on the alerts, as well as the physiological and symptom data from the Medly system [29].

#### Participants

English-speaking individuals living with heart failure were eligible to participate if they were current patients of the Medly heart failure program at Toronto General Hospital. People living with heart failure were recruited across a range of demographic factors to ensure representation with respect to age, sex, ethnicity, place of birth, highest education received, place of residence, living arrangement, and income in Canadian dollars. Efforts were also made to ensure that participants with varying levels of comfort using smartphones were included, acknowledging that recruitment from a smartphone-based remote management program might result in participants who are more inclined to access health care virtually.

All 13 people living with heart failure approached for the study agreed to participate. No individuals refused to participate or withdrew from the study. Likewise, all clinicians of the Medly program were also invited via email to participate in the study. After these initial interviews with clinicians of the program, snowball sampling was used to recruit additional clinicians and researcher participants. A total of 10 clinicians and researchers were approached for the study, 9 of whom agreed to participate. One clinician declined participation in the study due to their high workload associated with the COVID-19 pandemic.

Consistent with guidelines regarding qualitative research, the number of participants recruited was determined based on the principle of data adequacy, which prioritizes the ability of the data collected to provide a nuanced and rich understanding of the topic of study [30]. Data collection occurred until data adequacy was achieved, which was achieved at 23 participants.

#### Data Collection

Author AS, who had no prior relationship with the study participants, conducted one-on-one semistructured interviews in English with all participants. All interviews were conducted over the phone due to public health restrictions associated with the COVID-19 pandemic at the time of interviews and the wide geographical distribution of participants in the Medly program. The interviewer conducted the phone interviews in a private space, and all individuals who participated also did so in a private space at their home or workplace. No participants reported the presence of additional individuals at the time of their interviews.

At the time of data collection, AS was a PhD candidate in Health Informatics Research with formal training in qualitative methods and prior experience conducting qualitative studies.

Participants were made aware of AS’s role as a student. AS had previously worked in the community mental health sector, largely from a public health and patient-engagement perspective. These prior experiences with patient perspectives on mental health care and digital health research were important to consider when engaging with clinician participants in this study, whose perspectives were less familiar to AS. To determine how her experiences in community mental health and digital health research shaped the findings of the research, AS conducted memoing throughout the research process and engaged in discussions with the broader research team, which included clinicians from the disciplines of mental health nursing, psychology, and psychiatry.

Interviews ranged from 40 to 60 minutes in duration. Interview guides tailored to each participant type (people living with heart failure, clinicians, and researchers) were created with the research team. For people living with heart failure, the guide included open-ended questions and prompts inquiring about the mental health needs of participants based on their experiences living with heart failure and/or seeking mental health services (Multimedia Appendix 1). Participants were asked to share their perspective on current approaches to managing their mental health and their needs for new mental health service delivery models. Similarly, the clinician (Multimedia Appendix 2) and researcher (Multimedia Appendix 3) interview guides asked participants to draw upon their clinical or research experience when discussing their perspectives on current approaches to addressing the mental health challenges faced by people living with heart failure, difficulties with current approaches, and service delivery needs and requirements to address these challenges. Interviews were audio recorded, stripped of identifying details, and professionally transcribed verbatim. No repeat or follow-up interviews were conducted, nor were transcripts or analyses returned to participants for member checking purposes.

#### Data Analysis

Two study team members (AS and ASF) analyzed the data to offer different perspectives on the analysis. At the time of analysis, AS and ASF were both PhD candidates in Health Informatics with training and experience in qualitative research. AS came from a public health background, and ASF offered the perspective of a clinician as a nurse. First, both authors read and reread transcripts to gain an in-depth familiarity with the data. Second, each team member independently coded the data inductively and grouped them into initial categories and themes. NVivo (version 12; QSR International) was used to organize and code data. Third, following the development of the initial themes, AS and ASF met to discuss their codes and themes and to highlight where their analyses were similar and different. Where coding and themes differed, both team members discussed these differences to understand where each individual saw unique aspects of the analyses and their relevance to the research questions and phenomenon of study. These discussions were intended to deepen the analysis through the different perspectives and experiences of the authors rather than to achieve consensus on the coding and themes. Finally, both team members met to construct a final set of codes and themes based on their independent analyses. Both AS and ASF memoed throughout the analysis to reflect upon how their personal perspectives may have contributed to the analysis.

### Objective 2 (Methods)

As part of the second study objective to develop a virtual mental health service for people living with heart failure, prototypes of the virtual stepped care service components were developed and visualized through design mockups and a service blueprint. Consistent with a service design approach, the design of the virtual stepped care service components was conducted iteratively. The initially visualized prototypes of the virtual stepped care model were presented to relevant stakeholders and refined based on expert feedback. These included experts in psychiatry, psychology, program administrators of existing virtual health services, digital product design, hospital legal services, and nursing staff from the Medly remote management program. For example, in designing the depression screening component of the model, a validated screening tool was initially selected based on the literature. However, iterative consultation with psychiatrists, administrators, and hospital legal services highlighted the need for specific design features for the remote administration of the screening tool at the hospital. This prompted specific language to be incorporated in the Medly app to ensure clinical safety. After multiple rounds of stakeholder engagement and refinement, a final prototype of the virtual stepped care service was reviewed and vetted by a core expert group that included psychiatric, psychological, nursing, and health informatics perspectives.

### Ethical Considerations

This study was approved by the University Health Network Research Ethics Board (protocol nos 16‐5789 and 20‐6329) and the University of Toronto Research Ethics Board (protocol nos 40,274 and 41477). All participants provided written informed consent before study enrollment and were not provided with compensation for their participation in this study. Study findings are reported in alignment with the Standards for Reporting Qualitative Research [31].

## Results

### Participants

A total of 22 participants were interviewed, including 13 people living with heart failure and 9 clinicians and researchers specializing in mental health or heart failure care (Table 2).

**Table 2.** Demographic characteristics of people living with heart failure interview participants (N=13).

Characteristic	Participant, n (%)
Age (years)	
21-30	2 (15)
31-40	1 (8)
41-50	0 (0)
51-60	2 (15)
61-70	4 (31)
71-80	2 (15)
81-90	2 (15)
Sex	
Male	7 (54)
Female	6 (46)
Ethnicity	
White (Caucasian)	8 (62)
Black	1 (8)
Filipino	1 (8)
South Asian	1 (8)
Chinese	1 (8)
Arab or West Asian	1 (8)
Place of birth	
Canada	8 (62)
Other	5 (38)
Highest education achieved	
High school	3 (23)
Trade or technical training	2 (15)
College or university	5 (38)
Postgraduate	3 (23)
Place of residence	
Urban	6 (46)
Suburban	4 (31)
Rural	2 (15)
Not declared	1 (8)
Living arrangement	
Living with family or partner	9 (69)
Living alone	3 (23)
Living with friends or roommates	1 (8)
Income (CAD $)	
<15,000 (US $10,935.67)	1 (8)
15,000-49,999 (US $10,935.67-US $36,451.52)	3 (23)
50,000-74,999 (US $36,452.25-US $54,677.65)	7 (54)
>75,000 (US $54,678.37)	1 (8)
Not declared	1 (8)
Comfort with smartphone	
Very comfortable	2 (15)
Comfortable	2 (15)
Somewhat comfortable	3 (23)
Not comfortable	2 (15)
Does not use a smartphone	3 (23)
Not declared	1 (8)

### Objective 1: Strategies to Address Barriers to Accessing Mental Health Services for People Living With Heart Failure

The following section describes the themes and strategies to address health system barriers to mental health care access across each domain of Levesque’s framework [28].

#### Approachability: Collective Action to Find Signals of a Range of Distress

In response to the barriers to detecting mental health concerns among people living with heart failure, clinicians highlighted the need to work together to detect a range of distress states. Participants posited that this would be best achieved by leveraging the unique and complementary strengths of each type of clinician. Psychiatrists recognized that patients had a strong therapeutic alliance with their chronic disease clinician, such as the close nurse-patient relationship facilitated through remote patient management. As such, this relationship was thought to provide another layer of detection of distress, potentially sooner. Likewise, cardiac clinicians noted that they could benefit immensely from the structured and detailed assessment skills of mental health clinicians, for which they did not feel fully prepared or trained*.*

*We really rely on [non-psychiatric clinicians] to identify distress, to explain it to their patients. Because they really have the therapeutic alliance with their patients and can see it in action and can help us contextualize some of the nuances of what they’re seeing. They are seeing things that may be signals of more distress that they need us to help them discern. So, what is the mental health literacy of our health care community that provides very specialized and medical, and surgical interventions? And what is holistic care?*

[Clinician 1]

Clinician perspectives were complemented by patients who had an interest in holistic assessment of their health, recognizing that the current telemonitoring of their heart failure symptoms did not tell a full story of their health and sometimes painted a picture of well-being that they did not feel was accurate for their situation. More holistic and dynamic assessment of different aspects of health was not only seen as more accurate but also a way to humanize their care.

...statistically, I’m fantastic. And yet, I feel like a bag of sh*t, and I wish I wasn’t here...And I know they try to measure how you’re feeling, but if the measurement tools they’re using aren’t gaged to efficiently judge that, then what criteria are they using to determine whether or not you’re having a good ride or not?

[Patient 1]

#### Availability and Accommodation: Capacity Building While Staying in Your Lane

While acknowledging the constraints of the mental health care system, participants emphasized the need to respond to a range of distress that is possible in the context of physical health conditions, including subclinical distress. This involved expanding the “helpers” in the space who could recognize and respond to mental health concerns, thereby ensuring that psychiatrists were not the only clinicians providing support for this aspect of health.

*I think we’ve lost a little bit of that sense of the spectrum of who all of the helpers are in this space. No one thinks of your personal trainer as being a cardiac specialist prevention office. But effectively that’s what they are right? You’re going to the gym and your personal trainer to try and prevent this from becoming a disease. But it is not medicalised, right? Unless I have a cardiac event and then I go to cardiac rehab and I get the trainer because I’ve had an event. And then it’s becoming medicalised. Because we know it’s good but people aren’t going to do it on their own. Or they can’t afford to...I think that’s the challenge—who are all of these helpers in this space? And which ones need to be medicalised within the program or not?*

[Clinician 1]

Building the mental health literacy of both patients and clinicians was thought to facilitate a wider range of helpers to draw upon, instead of assuming that all had adequate mental health literacy at the start of seeking care. Participants noted that this education could include understanding the psychosocial aspects of heart failure, normalizing distress when living with heart failure, and knowing the mental health services available. In addition to these areas, the ability to discuss the intersections between mental and physical health was also noted as an especially important training area for all clinicians.

*That [mental health] literacy, that education for patients and their families, their caregivers, and for the other health care professionals… All of that shared understanding of what this is in that space that you’re trying to accomplish, and what’s actually available. Some basic competencies and taking space for patients to engage in it. Whatever we decide that common understanding is.*

[Clinician 1]

In addition to improving mental health literacy among patients and non–mental health clinicians, improving the understanding of heart failure support, such as the Medly program, was also highlighted as an important training area for mental health care professionals, thereby fostering greater mutual understanding between physical and mental health clinicians.

Balanced with the need to expand participation from nonmental health clinicians was also the desire and need to “stay in your lane” or remain within one’s areas of expertise. While patients and clinicians believed that integration of mental and physical health care was needed, some level of separation of each type of care was also valued. For clinicians, this meant clear clinical pathways and a preference for applying a referral-based approach to mental health concerns, whereby heart failure clinicians could refer out mental health concerns among their patients and were not deeply involved in the ongoing mental health care of their patients.

*Cardiologists are not going to want to be dealing with mental health issues in terms of management. Identification? Absolutely. Appropriate referral? Hell yes. Management? No way.*

[Clinician 2]

For patients, maintaining a level of separation between their mental and physical health care meant a desire to keep the details of their mental health care separate from their heart failure clinicians, while still ensuring that clinicians were broadly aware of any care received that might affect their cardiac care.

*I think all your health care providers should be on top of you in the metaphoric sense that they need to know what’s going on in your life at all times. Because if there’s something that is off and my GP hasn’t noticed, well, I feel like my cardiologist should or my endocrinologist should. There should be some level of collective communication going on behind me that, OK, they can access it and they have some idea that this is what’s going on…I would want [my cardiologist] to be [aware of my mental health]. And if they’re not, I would call them myself and tell them.*

[Patient 2]

#### Affordability: Working With the Underinsurance of Mental Health Care

A central element of the model of care noted by participants was to work within the limited mental health resources and supports available to reserve publicly funded psychiatric services for the most complex and severe cases. Recognizing these constraints, 1 clinician discussed the benefits and challenges of proactive (engaging mental health supports early to reduce risk) and reactive (awaiting mental health concerns to be referred to a mental health clinician) models of care. Leveraging existing mental health services and supports as much as possible, instead of developing new ones, was considered the most feasible mode of care.

*...there are so many gaps in mental health care. And not an organized system provincially. So trying to do what we can, but also knowing that we can’t boil the ocean, right? There’s just so much need, and being able to draw some of the boundaries in that is going to be a bit of a challenge for us. So we’re trying to be careful not over-promising what we can deliver to patients and their families.*

[Clinician 1]

#### Appropriateness: Personalization, Choice, and Quality Connections

Patients desired a quality connection to mental health care services tailored to their individual needs, preferences, and readiness. Inherent to finding such connections was the ability to have various options to choose from and to create opportunities for patient choice. Despite the desire for choice, patients noted that the current health care system did not allow for such an approach. One patient described their ideal system, which would offer the mental health care they were seeking.

*...health professionals would address you as if you were a real person with a real life and they had some idea of what that life was, what their job was, and what they were protecting when they were looking after you.*

[Patient 3]

Foundational to a quality connection was the existence of mental health service options to choose from, as well as different delivery method options for each service that went beyond the in-person and virtual dichotomy (in-person, phone, videoconferencing, and asynchronous). This was echoed by clinicians, who noted that a multipronged approach was needed for quality mental health support among those living with heart failure, such as a combination of psychotherapy and medication.

*I think truly the answer is to have a suite of opportunities that are therefore suited to each patient on a more individual basis as opposed to sending everybody for one plan.*

[Clinician 2]

*...you can’t just give a drug. You’ve got to counsel as well. You’ve got to advise about self-care. You’ve got to link them up with other people. So it’s a multi-pronged approach where you’re talking about the complex clinical level patient.*

[Clinician 3]

Once options were presented to patients, they desired to participate in the selection of the appropriate service for them in partnership with their clinician, as well as having an option to withdraw from mental health care at any time. When selecting the right service, patients considered their personal technology use and digital barriers, stage in chronic disease course, and prior experiences with mental health care through themselves or a loved one. In addition, mental health clinician factors, such as age and gender, were also important to them. Patients highlighted that recognizing the uniqueness of each individual’s situation was an important goal when seeking mental health care, even among those experiencing similar health circumstances.

*It’s not textbook either. So, you can have five [people like me] in a row. We all have similarities, but we’re all very different. And we have different needs for tools...it’s leaving it up to the individual where their boundaries lie…you can pick and choose what tools apply to you.*

[Patient 4]

#### Foundation Principle 1: Reducing Treatment Burden

A central theme raised by all participants was the importance of reducing the burden of treatment on clinicians and patients. For clinicians, this arose through the emphasis on managing the workload of nurses. While leveraging the strong nurse-patient relationship established through remote management was considered a valuable opportunity to detect and respond to mental health concerns, there were simultaneous concerns that increasing engagement with mental health would contribute to nurse burnout. To address this, engaging nurses with clear expectations and workflows was strongly recommended by nurse and cardiologist participants.

Similar to the nurses, cardiologists expressed interest in their patients’ mental health needs, but noted a need for a narrow role in the care model due to their limited capacity and time. Applying a referral-based approach or automatic enrollment to virtual mental health supports to reduce the reliance on cardiac clinicians for patient access to mental health care was one strategy proposed to improve the feasibility and acceptability of the care model. Similarly, patients interviewed saw this approach as making mental health care more approachable and less intimidating.

*...maybe patients could access it themselves… I wonder if it was like something that they said “you can check out this extra thing if you want, on your own time.” Something that is a little bit more passive rather than me directing them to it.*

[Clinician 4]

Likewise, patients expressed motivations to reduce the work associated with managing their conditions and were keen to understand the clear benefits of mental health support before adopting further treatments. When discussing potential mental health screening approaches, patients described a desire to avoid unnecessary work by completing readings only when they were struggling.

*I think it could be optional because basically when I’m fine I don’t really want to spend time filling out extra stuff around my mental health. But you know I want the option where I can click ‘not applicable’ or something.*

[Patient 5]

Contrary to common notions that self-guided supports are lower intensity, some patients perceived mental health supports that involved heavy amounts of reading as more work-intensive than those relying on video or speaking to someone.

*...giving them something to read is a lot of time and commitment toward something, or if you’re making them read it then make it short and that people’s attention spans are very short these days.*

[Patient 6]

#### Foundational Principle 2: Building Upon Organizational and Programmatic Strengths

Building upon organizational strengths was thought to be an important approach to improve access to mental health care. At the hospital, the organization had several strengths that promoted interest and buy-in for an initiative focused on improving access to mental health care for people living with heart failure, including a corporate strategy outlining integration of mental and physical health care as a priority, as well as examples of chronic disease clinics that provided strong mental health support (eg, cancer care). These were noted as strengths for the organization to build upon, generating enthusiasm and interest in expanding further within heart failure care. In addition to broader organizational strategies, participants noted opportunities to build upon the strengths of the heart failure telemonitoring program itself, namely its symptom monitoring, communication to the patient’s care team, strong nurse-patient relationship, and already high use of the Medly app by patients. Leveraging the Medly app in new ways was thought to promote trust in addressing the new topic of mental health, which could potentially spill over into other virtual mental health interventions that patients are connected to as a result of the model of care.

*I feel that patients trust Medly a lot. So maybe having mental health support within it, being accessible from it, might be an added element of trust to it.*

[Clinician 4]

### Objective 2: Virtual Mental Health Service Design for a Heart Failure Remote Management Program

Based on the strategies identified through qualitative interviews, the following section addresses the second objective of describing the design of a virtual mental health service for a heart failure remote management program. Table 3 summarizes how the service design components were tailored directly to the strategies identified for Objective 1.

**Table 3.** Strategies and features of the virtual mental health stepped care model to address barriers faced by people living with heart failure when accessing mental health services.

Domain of access	Health system barriers [6]	Strategies	Virtual mental health stepped care features
Approachability	Difficulties detecting mental health concerns; unpreparedness for referral conversations; and denial, stoicism, and self-reliant coping methods.	Collective action to find signals of a range of distress	Integrating mental health into virtual chronic condition management programs.
Availability and accommodation	Inconsistent pathways to mental health services and the inconvenience of in-person delivery.	Capacity building while staying in your lane	Referral-based approach; clear workflows; preserving separation of mental health care pathways from heart failure; and virtual care delivery.
Affordability	Limited human resources due to underinsurance of mental health care, lack of full insurance coverage, and high cost of psychological services.	Working with the underinsurance of mental health care	Integration of existing mental health services and use of no-cost mental health services.
Appropriateness	Insufficiency of generic mental health services.	Personalization, choice, and quality connections	A suite of mental health service options, choice at certain stages of the stepped care model, and tailored mental health supports for heart failure.
Foundational principle 1: reducing treatment burden	—^a^	—	—
Foundational principle 2: building upon organizational and programmatic strengths	—	—	—

a Not available.

#### Approachability: Medly Mental Health Module

**Figure 1.**
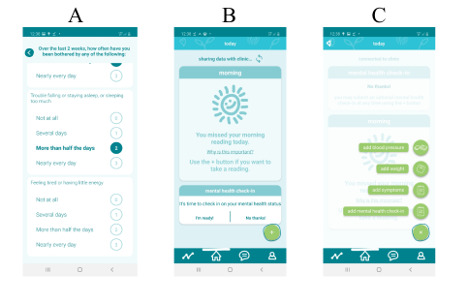
Design of the depression screening module for the Medly smartphone app for heart failure remote management.

Although several heart failure guidelines recommend the regular screening of depression, this practice has not yet been conducted within heart failure care at Toronto General Hospital [1,3-5]. To promote the approachability of mental health services, screening for depressive symptoms via the 9-item Patient Health Questionnaire (PHQ-9) was integrated into the existing Medly telemonitoring smartphone app (Figure 1A), which requires patients to report their weight, blood pressure, heart rate, and symptoms daily [29].

The PHQ-9 is a commonly used questionnaire to screen for symptoms of depression, with each item corresponding to a criterion for depression as outlined in the *DSM-IV* (*Diagnostic and Statistical Manual of Mental Disorders* (Fourth Edition) [32]. Scores on the PHQ-9 can range from 0 to 27, with specific score ranges corresponding to mild (5-9), moderate (10-14), moderately severe (15-19), and severe (20-27) depressive symptoms [32]. Prior research has validated the use of the PHQ-9 among people living with heart failure [33]. Meta-analytic research has identified a cutoff score of ≥10 to diagnose clinically significant symptoms of depression with high sensitivity (80%) and specificity (92%) in medical settings, including cardiology [34].

In an effort to preserve patient choice, the PHQ-9 screening module was intentionally made optional, allowing patients to decline to participate in a screening, described as a “mental health check-in” in the app (Figure 1B). As the heart failure portion of the Medly app is intended for daily use and the PHQ-9 inquires about the depressive symptoms experienced over the past 14 days, the PHQ-9 screening module was set to prompt patients to complete screening every 14 days from their last reading, with the option available for patients to engage in more frequent screening through an “add mental health check-in” option (Figure 1C).

Once PHQ-9 screening is completed by the patient, the module was designed to provide patients with an automated alert based on the PHQ-9 score range. This approach, involving self-report screening followed by an algorithm-based interpretation and alert, aligns with the existing app design for heart failure previously described in the “Methods” section [29]. To coordinate alerts between mental health and physical health and to reduce confusion for patients, a “single severity system” was developed, which assigns and presents a single alert to the patient’s state based on heart failure, mental health, or both symptoms. Alert states are visible immediately to the patient after completing their reading, as well as to the clinician via email and the clinician’s dashboard for notification and review.

In addition to an alert state, self-care messages are automatically provided to the patient based on their PHQ-9 score. Each automated message notifies patients of their score, provides the interpretation of their score (eg, mild, moderate, severe depressive symptoms, self-reported thoughts of harm to self or others), and suggests next steps based on their score (eg, call your Medly nurse). Importantly, as the ninth item of the PHQ-9 pertains to suicidal ideation, language for this self-care message was taken from institutional guidance on the remote administration of the PHQ-9 and provides crisis resources and information in a “Learn More” section of the Medly app. A suicide risk management protocol was also developed for instances where a patient self-reports thoughts of harm to self or others on the PHQ-9, which outlined how to connect patients to an on-call psychiatrist.

#### Availability and Accommodation: Referral-Based Workflows and Virtual Delivery

**Figure 2.**
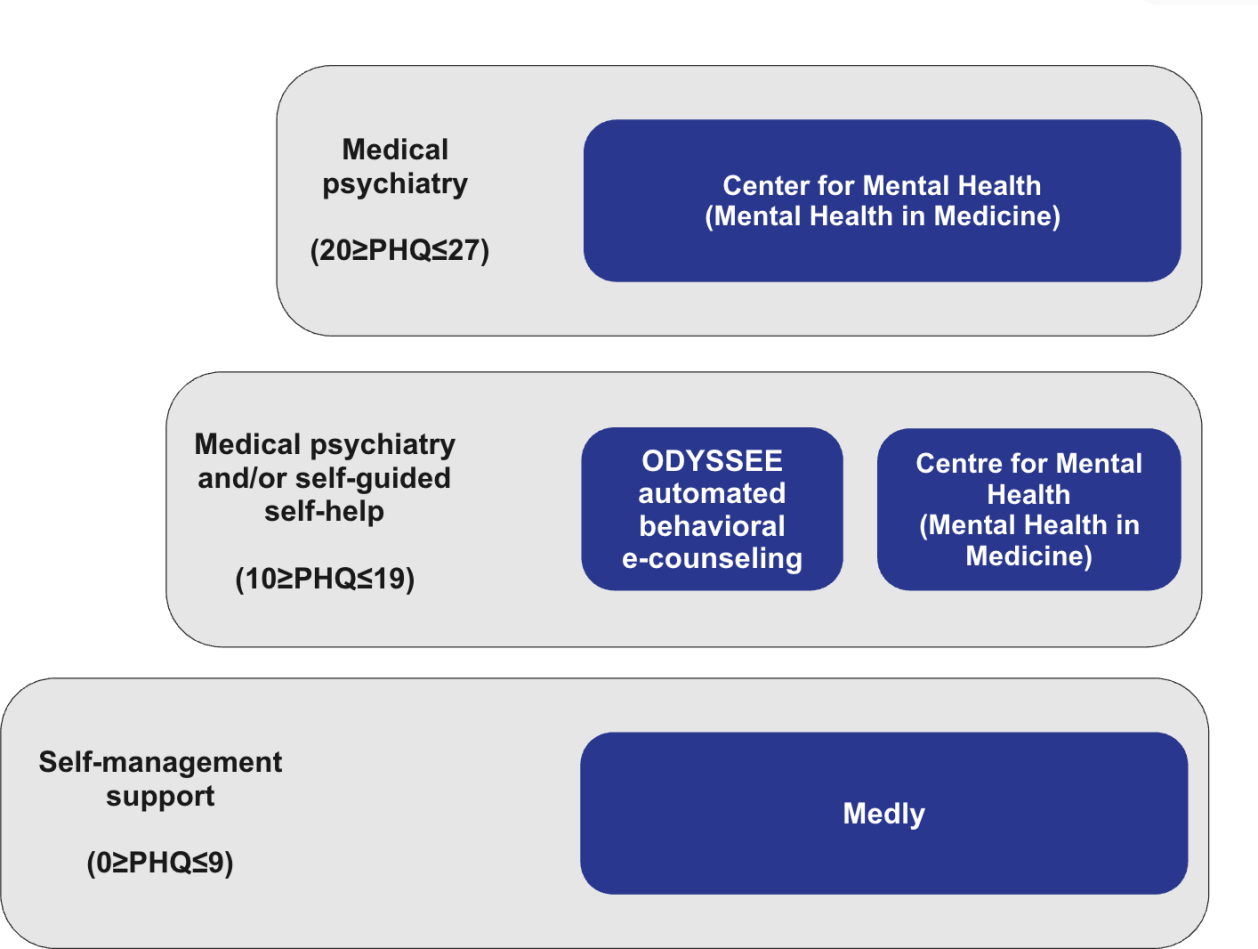
Virtual mental health stepped care model for the Medly heart failure remote management program. PHQ: Patient Health Questionnaire.

Upon screening for depressive symptoms, a workflow for clinicians to follow up with patients for clinically significant symptoms of depression (PHQ-9≥10) was identified. A service blueprint was created to visualize the pathways between screening and various care options. After an alert, patients who report clinically significant symptoms of depression are referred to a mental health nurse practitioner at a specialized medical psychiatry clinic called the University Health Network Mental Health in Medicine Clinic, where they receive further assessment to determine whether psychiatric care is needed [35]. Should the mental health nurse identify no need for psychiatric care (moderate depressive symptoms and no suicidal ideation), patients are provided with the option to choose from 2 existing mental health services: virtual mental health care via the ODYSSEE automatic behavioral e-counseling platform (Behavioural Cardiology Research Unit, University Health Network) [36] and usual care at the Mental Health in Medicine Clinic (Figure 2) [35]. Patients with nonclinically significant symptoms of depression on the PHQ-9 (score between 0 and 9) are not provided with additional interventions and are advised to continue using the Medly remote monitoring app to self-manage their condition.

In drafting the service blueprint with stakeholders from the ODYSSEE team and the Mental Health in Medicine Clinic, the registration or referral processes required to access these services were considered to ensure the patient experience was intentionally understood, and potential pain points were identified and minimized, where possible. Through this service design approach, the different systems used by each of the care options were identified as a potential pain point, as they would require patients to create multiple account logins to proceed with a referral. For example, in the Medly program, patients are given account credentials to log into their Medly app. Should a patient in the model of care choose to access the ODYSSEE automated behavioral e-counseling, they would need to create separate account credentials to access this care option. While it was not feasible to reconcile this into a single login at the time of service design, the service design process helped identify this as a pain point to potentially address in the future.

#### Affordability: Use of Existing Mental Health Services

Clinical champions of each service (ODYSSEE and the Mental Health in Medicine Clinic) who shared clinical and research interests in improving access to mental health care for people living with heart failure were identified for this project. ODYSSEE and the Mental Health in Medicine Clinic were selected to be included in the model of care as they already existed within Toronto General Hospital and offered content or care that was tailored to the journey of living with heart failure, which was among the factors that people living with heart failure previously had identified as important to quality mental health care [6]. Importantly, both care options are available free of charge to the patient, allowing patients to select mental health services without restrictions based on income or private insurance coverage. Services at the Mental Health in Medicine Clinic are covered under the universal provincial insurance plan, while the ODYSSEE program was covered through research funding at the time of the research. The inclusion of ODYSSEE was considered to be especially important to promote the affordability of mental health services, as it provides psychoeducational content based on principles of cognitive behavioral therapy. As access to publicly funded psychological services is highly limited in Canada, the introduction of ODYSSEE into the service delivery model served to make psychological services and content more readily available to people living with heart failure.

#### Appropriateness: Patient Choice

Should patients’ PHQ-9 score range fall within 10‐19, and they are not found to require psychiatric care based on the assessment by the mental health nurse practitioner at the Center for Mental Health, the model encourages patient choice by allowing them to choose from the ODYSSEE automated behavioral e-counseling program or standard care from the Mental Health in Medicine Clinic. The element of patient choice at this stage was intended to allow patient preferences surrounding mental health care to be integrated into the model of care, where possible. Patients with a PHQ-9 score between 20 and 27 are not offered choice in their intervention and are guided to care as usual via the Mental Health in Medicine Clinic.

## Discussion

### Principal Findings

In this study, we describe the design of a virtual mental health stepped care service for people living with heart failure enrolled in a heart failure remote management program in Ontario, Canada. Building upon previous research that documented barriers to accessing mental health services for this population, we explored strategies to mitigate these barriers and incorporated them into a new clinical service [6]. The contributions of this study lie not only in the design of the virtual stepped care model, but also in how stakeholders and existing clinical resources in the implementation context were engaged to conceive this new service.

Through a design process involving the perspectives of people living with heart failure, clinicians, and researchers, the resultant virtual mental health service includes a depression screening module within the existing Medly smartphone-based heart failure telemonitoring app to improve detection of depressive symptoms. Following screening, referral-based pathways were defined to replace previously inconsistent patient pathways to mental health services. To mitigate affordability barriers, existing mental health services available within the implementation context were selected to allow people living with heart failure to access mental health care without incurring out-of-pocket costs. For those found to be experiencing moderate scores of depressive symptoms, the stepped care service offers people living with heart failure the ability to choose between automated behavioral e-counseling and usual care through the hospital’s medical psychiatry clinic [37]. The goal of this newly created intervention is to provide more holistic heart failure care that addresses both patients’ physical and mental health needs to improve the person-centeredness of heart failure care and, consequently, the quality of care received.

An emerging yet growing body of research has argued that stepped care models should be context-specific and designed based on principles, rather than seeking to apply a standardized model across settings with different mental health resources [22,38]. Our study aligns with this notion and offers an example of how stepped care services can leverage existing mental health resources within an organizational context that were not initially designed for integration, which is most often the case in real-world settings. While we anticipate that the context-informed design approach used will promote the feasibility of implementation of the model in subsequent phases, applying such an approach also presents limitations in this research. Specifically, while attuning to the needs and existing capacities of the implementation context when designing the service delivery model may promote its feasibility of implementation, it may also prevent a more comprehensive approach to care that requires more intensive services to meet the wide range of needs of the intended population. For example, despite the intention and desire for more person-centered approaches to mental health service delivery for the heart failure program, stakeholders engaged in this study called for a more traditional, referral-based model of stepped care instead of more contemporary and flexible versions such as Stepped Care 2.0 [20,21]. This design decision reflects the trade-offs between designing theoretically robust interventions and those that are practically implementable based on the readiness and capacities of an organization.

In addition to the practical design of the virtual stepped care service, our findings offer conceptual contributions to the growing stepped care literature that can help inform similar undertakings in other contexts. Recently, stepped care scholars have proposed that “patient readiness to engage in care” is a key principle underlying treatment allocation within person-centered variations of stepped care (eg, Stepped Care 2.0) [20,38]. While participants in this study affirmed the importance of considering their readiness to access mental health care (eg, desire to revisit potentially difficult emotions and experiences, their comfort engaging with long-term mental health care), they also highlighted a nuance not yet discussed in the stepped care literature. Participants considered the work and burden of engaging in mental health care in their decisions to access mental health services, especially in light of their treatment for heart failure and other chronic conditions they were already engaging in (eg, tests, medications, hospitalizations, medical appointments, and complex self-care behaviors). The impact of such “illness work” on the patient, per sociological perspectives from Corbin and Strauss [39,40], is known as treatment burden*.* Emerging research indicates that digital health interventions may also affect treatment burden, both positively and negatively, by at times introducing illness work [41]. In this study, this was evident as people living with heart failure viewed commonly regarded “low intensity interventions,” such as educational modules, as “too much work” when education was delivered in a traditional text-based format. These findings suggest that the work of digital mental health interventions must be considered in the stepped care allocation process, especially those intended for people living with heart failure. Stepped care models may find it fruitful to encourage patients to not only connect with the intervention that aligns with their readiness but also consider the amount of illness work they can adopt, given their total workload. Further research is needed to investigate the concept of treatment burden as a principle guiding treatment allocations within stepped care.

While this study focused on barriers at the health system level, it is important to note that our research team previously documented barriers at the patient level, including low mental health literacy, stigma, difficulties attending services in person, and lack of insurance coverage to pay for psychological services [6]. Although the intervention components described in this study may also reduce patient-level barriers, more substantial efforts may be needed to fully address these barriers. For example, we anticipate that the inclusion of screening in the virtual stepped care service will not only improve the approachability of the health system but also enhance patients’ ability to perceive their mental health needs. Moreover, the focus on using existing mental health services as part of the intervention, instead of creating new ones, was to improve the affordability of mental health services, which may also circumvent insurance barriers at the patient level. Future studies should explore, incorporate, and evaluate complementary interventional strategies to address patient-level barriers to accessing mental health care alongside the health system–level strategies identified in the current research.

### Comparison With Prior Research

While the exploration of virtual stepped care services in heart failure care is limited, studies in other clinical areas offer examples of how these interventions have been designed. Indeed, previous studies describing the creation of virtual stepped care services for other health areas appear to suggest that the engagement of interdisciplinary stakeholders, such as clinicians, patients, researchers, and technology developers, is critical to the design of these interventions and the evaluation of these service delivery models [42-48]. In this study, our team adopted a service design approach combining interviews and iterative feedback cycles, whereas other studies have commonly used design workshops [43,45,48] or focus groups [47]. While these exploratory qualitative approaches used in previous studies to empathize with the end users’ needs mirror those used in this study, some studies followed the design with user testing to further identify pain points and refine the intervention before implementation [43]. As user testing was not conducted during this study, usability challenges may impact the acceptance and use of the stepped care model and warrant consideration in the future.

A related strand of the literature has focused less on user-centered design and more on the practical realities of designing stepped care models in real-world settings [46,48]. These studies describe the organizational work required to establish partnerships, create shared platforms, map existing services, define levels of care and patient pathways, and deliver the intervention under a unified brand by forming advisory or working groups composed of interdisciplinary stakeholders [46]. Both the findings of this study and previous studies highlight the need to balance organizational collaboration with patient engagement activities to align various stakeholder interests with a clear goal of defining an improved patient journey.

### Limitations

The study findings should be interpreted in light of the following limitations. First, participants were recruited and interviewed when COVID-19 physical distancing and stay-at-home orders were implemented. The strategies integrated into the virtual stepped care model may therefore represent a period when virtual care was more strongly preferred and used. Further research is needed to explore the acceptability of this service delivery model when COVID-19 measures are no longer in place [49]. Second, while participants were purposively recruited across a range of demographic factors, the sample interviewed was English-speaking people living with heart failure with access to a heart failure remote management program through a large urban academic hospital. As such, the strategies to improve access outlined in this study may not represent the full range of ways to address barriers faced by people living with heart failure and may be most transferable to people living with heart failure with higher levels of health care access. Future research is needed to determine appropriate strategies for people living with heart failure with lower levels of health care access and English proficiency. Health service designers, researchers, and policymakers seeking to improve access to mental health services for people living with heart failure may find it valuable to engage in a similar process of engaging diverse stakeholders to define strategies most suitable for their local context.

### Conclusion

Amid growing interest in stepped care models to implement virtual mental health technologies, this study outlines how virtual stepped care services can be designed and tailored to their intended implementation context to address health system barriers to mental health care. In addition to identifying strategies that heart failure remote management programs may integrate to improve access to mental health care for people living with heart failure, the findings of this study offer a practical approach to designing virtual stepped care models in partnership with local stakeholders that is both theoretically informed and rooted in the needs and capacities of the implementation context. Through this process, two key principles were identified for virtual stepped care services designed to suit their local contexts: (1) building upon organizational and programmatic strengths, and (2) reducing treatment burden. While the specific components of the virtual stepped care service may be most applicable to similar settings in urban, high-resource environments, the theory- and context-informed service design approach can serve as a process for future initiatives to improve access to mental health care for people living with heart failure. Future research should explore the feasibility of applying this design approach in different settings, as well as investigate the applicability of treatment burden and building upon contextual strengths as broader principles guiding virtual stepped care models.

## Supplementary material

10.2196/82139 Multimedia Appendix 1 People living with heart failure interview guide.

10.2196/82139 Multimedia Appendix 2 Clinician interview guide.

10.2196/82139 Multimedia Appendix 3 Researcher interview guide.

## Abbreviations

*DSM-IV*: *Diagnostic and Statistical Manual of Mental Disorders* (Fourth Edition)
PHQ-9: 9-item Patient Health Questionnaire
