# Supplementary material for: Development of Virtual Mental Health Stepped Care Service for a Heart Failure Remote Management Program: Qualitative Descriptive Study
Source: JMIR Form Res. 2026 Apr 14;10:e82139. doi: 10.2196/82139 (PMC13078668; doi:10.2196/82139)
Supplement: Multimedia Appendix 1 [file formative-v10-e82139-s001.pdf]

1. Could you tell me about the conditions that you live with?

2. Can you tell me a little bit about what your health journey has been like since you have been diagnosed with heart failure?

3. From your perspective, what does it mean to have good mental health when living with heart failure? What role do you think you play in this, if any?

4. How has your journey with heart failure impacted your mental health? For example, are there times that you have felt stressed, or where it has impacted your emotions, behaviour, or relationships with others?
    a. During these times, what helped you know that your mental health was affected?

5. How do you generally manage your mental health, if at all?

6. Who have you talked to about your mental health, if anyone? (professional or informal)
    a. Have you ever talked with a healthcare provider about your mental health?

7. Are there any mental health services or support you are aware of? How did you come to learn about them?

8. What role has your heart failure clinicians played in helping you find mental health care services?
    a. If they have not played a role, what role would you have wanted them to play in helping you find mental health services?

9. If you have accessed mental health services, what has been your experience trying to access mental health care services?
    a. What was your experience like…
        i. Traveling to the service?
        ii. Location/format of these services?
        iii. Booking an appointment (if applicable)? Once you booked an appointment, how long did it take for you to connect with a mental health professional (if applicable)?
        iv. Hours of operation/support of the service?

10. Were there any financial costs involved in any of the mental health services you accessed? If so, how did this affect your journey of seeking mental health care?

11. What has been the quality of care you received from mental health professionals?

12. Do you feel your mental health needs were met through this care? If not, what do you feel was missing to improve your experience?
13. Right now, Medly asks you to input your physical health symptoms every day and communicates it to your care team. Would you want Medly to periodically ask questions about your mental health?

14. If the Medly program were to offer you access to mental health supports, what types of supports would you be interested in using?

15. Is there anything else you would like to share about your journey with your mental health when living with heart failure that we haven't touched upon today?
