# Supplementary material for: Development of Virtual Mental Health Stepped Care Service for a Heart Failure Remote Management Program: Qualitative Descriptive Study
Source: JMIR Form Res. 2026 Apr 14;10:e82139. doi: 10.2196/82139 (PMC13078668; doi:10.2196/82139)
Supplement: Multimedia Appendix 2 [file formative-v10-e82139-s002.pdf]

1. From your perspective, what does it mean for people to have good mental health when living with heart failure? What role do you think you play in this, if any?

2. What are some of the common impacts of heart failure on mental health that you are aware of based on your clinical practice?
    a. While each patient's journey with heart failure may be different, are there specific stages or events in an individual's health journey that you notice mental health is often affected?

3. How do you typically identify when a Medly patient's mental health has been negatively affected? For example, do you conduct any screening, formally or informally? Where, when, by who, and what is this information used for?
    a. Can you recall an example of when a patient discussed their mental health with you? How do these conversations typically go?

4. What approaches, formal or informal, do people living with heart failure use to manage their mental health, based on your clinical practice?
    a. What role do you play in this, if any?

5. If you suspected that a Medly patient's mental health has been negatively affected, what are the current approaches to managing this?
    a. Have you referred patients to mental health services? If so, which ones? If not, what are some of the contributing factors to this?
        i. Can you recall any particular examples where you referred a Medly patient to a mental health service? How do these conversations typically unfold?
        ii. What do you find helpful or challenging about these conversations or processes?

6. Can you describe the booking process for Medly patients to access mental health services?
    a. From your perspective/understanding how accessible is it for patients to make appointments (if applicable), and how long does it typically take for them to connect with a mental health professional once an appointment is booked?
    b. Based on your clinical practice, are there specific aspects of the mental health services you refer to that help or hinder access to these services. For example, what are your thoughts on their location, format, and hours of operation, etc.?
    c. Can you recall any examples of Medly patients accessing mental health services and what their experience was like?

7. How affordable do you believe mental health services are to Medly patients?

8. From your perspective, what constitutes quality mental health services for people living with heart failure? To what degree do you feel these elements are present or not present in the mental health care available to patients in the Medly program?

    a. Can you describe an example of when a patient received high or low quality mental health care?

9. Do you feel that patients in the Medly program are having their mental health needs met? If not, what improvements do you see are needed?

10. Right now, Medly asks patients to input their physical health symptoms every day and communicates it to their care team. Would you want Medly to periodically ask patients questions about their mental health?
    a. What kinds of questions or information would you like to gather?
    b. Who would you want to be receiving and reviewing the data? What could that process look like?

11. If the Medly program were to offer patients access to mental health supports, what types of supports would you ideally like to see offered? Why?
    a. Are there any existing mental health supports you are aware of that could be offered or incorporated?

12. Is there anything else you would like to share about your observations or experiences supporting the mental health impacts people living with heart failure face, and their journey accessing mental health services?
