# Supplementary material for: Development of Virtual Mental Health Stepped Care Service for a Heart Failure Remote Management Program: Qualitative Descriptive Study
Source: JMIR Form Res. 2026 Apr 14;10:e82139. doi: 10.2196/82139 (PMC13078668; doi:10.2196/82139)
Supplement: Multimedia Appendix 3 [file formative-v10-e82139-s003.pdf]

1. From your perspective, what does it mean for people to have good mental health when living with heart failure? What role do you think you play in this, if any?

2. What are some of the common impacts of heart failure on mental health that you are aware of, based on your research?
    a. While each patient's journey with heart failure may be different, are there specific stages or events in an individual's health journey where mental health is often affected?

3. What are some common or best practices to identify when a patient living with heart failure is experiencing negative impacts on their mental health? Ideally, how should these conversations typically go?

4. What approaches, formal or informal, do people living with heart failure use to manage their mental health, based on your research?
    a. From your perspective, what role do clinicians play in this, if any?

5. If a clinician suspected that a Medly patient's mental health has been negatively affected, what are the current approaches to managing this?

6. Can you describe the booking process for Medly patients to access mental health services?
    a. From your understanding how accessible is it for patients to make appointments (if applicable), and how long does it typically take for them to connect with a mental health professional once an appointment is booked?
    b. Based on your research, are there specific aspects of the mental health services you refer to that help or hinder access to these services. For example, what are your thoughts on their location, format, and hours of operation, etc.?

7. How affordable do you believe mental health services are to Medly patients?

8. From your perspective, what constitutes quality mental health services for people living with heart failure? To what degree do you feel these elements are present or not present in the mental health care available to patients in the Medly program?

9. If the Medly program was to incorporate mental health support for patients, what features or interventions would you suggest incorporating?
    a. Are there new features or interventions you would suggest or are aware of from research?
    b. Are there existing interventions you are aware of that could be incorporated?

10. Is there anything else you would like to share about your observations or experiences researching the mental health impacts people living with heart failure face, and their journey accessing mental health services?
